# Influence of Mushroom Polysaccharide, Nano-Copper, Copper Loaded Chitosan, and Lysozyme on Intestinal Barrier and Immunity of LPS-mediated Yellow-Feathered Chickens

**DOI:** 10.3390/ani10040594

**Published:** 2020-04-01

**Authors:** Qiuli Fan, K. F. M. Abouelezz, Long Li, Zhongyong Gou, Yibing Wang, Xiajing Lin, Jinling Ye, Shouqun Jiang

**Affiliations:** 1 State Key Laboratory of Livestock and Poultry Breeding, Key Laboratory of Animal Nutrition and Feed Science in South China, Ministry of Agriculture and Rural Affairs, Guangdong Key Laboratory of Animal Breeding and Nutrition, Guangdong Public Laboratory of Animal Breeding and Nutrition, Institute of Animal Science, Guangdong Academy of Agricultural Sciences, Guangzhou 510640, China; fanqiuli_829@163.com (Q.F.); abollez@aun.edu.eg (K.F.M.A.); leeloong1985@sina.com (L.L.); yozhgo917@163.com (Z.G.); wangyibing001@gmail.com (Y.W.); linxiajing728@sohu.com (X.L.); YEJL2014@163.com (J.Y.); 2 Department of Poultry Production, Faculty of Agriculture, Assiut University, Assiut 71526, Egypt

**Keywords:** feed additives, intestinal immunity, growth performance, nano-nutrients, antibiotic alternatives, growing chickens

## Abstract

**Simple Summary:**

Recently, there has been an increasing worldwide interest in limiting or banning the use of synthesized antibiotic growth promoters used in animal production, so as to avoid the development of the drug resistance of human pathogens. This is accelerating the efforts of scientists to present new, natural and safe antibiotic alternatives that are adequate for animal and poultry production. This study contributes by evaluating some potent antibiotic alternatives in growing yellow feathered chicks, including mushroom polysaccharides, nano-copper, copper loaded chitosan, and lysozyme, versus a common antibiotic (Aureomycin). The dietary addition of the tested substances has demonstrated several beneficial effects, particularly in the case of polysaccharides (100 mg/kg) and lysozyme (500 mg/kg), on jejunal barrier, and immunity of yellow-feathered chicks aged 4–24 days, with comparable effects to the antibiotic treatment.

**Abstract:**

This study investigated the influence of dietary supplementation with some antibiotic alternatives on growth performance, intestinal barrier, and immunity of lipopolysaccharide (LPS) challenged chicks. Wenshi females, aged 4 days, were allocated randomly into eight groups, each with six replicates of 20 birds (n = 120/treatment), which received a basal diet supplemented with 0 (control), 0 (LPS), 200 mg/kg aureomycin, 50 mg/kg mushroom polysaccharide, 100 mg/kg mushroom polysaccharide, 500 mg/kg nano-copper, 300 mg/kg copper loaded chitosan, and 500 mg/kg lysozyme for 21 days. On day 18 and 20, the control birds were injected with 0.5 mL saline solution, the other treatments were injected with 0.5 mL saline containing 500 µg LPS/kg body weight (BW). The results indicated that LPS treatment reduced the BW, average daily gain (ADG), and daily feed intake (ADFI) than the controls (*p* < 0.05), and the antibiotic and the tested alternatives could not retrieve the normal BW, ADG, and ADFI. The tested additives reduced several negative effects of LPS; they reduced *diamine oxidase* activity and inflammatory mediators in plasma, jejunal mucosa, spleen and thymus, increased content of immunoglobulin in plasma and jejunal mucosa, and decreased gene expression of *inducible nitric oxide synthase* and *Cyclooxygenase 2* in jejunal mucosa.

## 1. Introduction

Lipopolysaccharides (LPS) are the major elements of the cell walls of gram-negative bacteria; they are endotoxins, which cause a strong response in normal animal immune systems and have been used in inducing immune stress in animal models [1]. LPS activates monocytes/macrophages to secrete various inflammatory cytokines [2], stimulates microglia, and decreases glutamatergic transmission that leads to memory deficits [3]. LPS also damages the intestinal barrier function [4], restricts the expression of innate immune receptors in intestinal epithelial cells [5], and enhances neutrophilic lung inflammation and pulmonary edema [6]. Antibiotics have been reported to counteract a variety of problems caused by LPS [7,8,9]. Most synthesized antibiotics, however, are potentially unsafe because they may increase drug resistance in human pathogens [10]. To avoid this negative influence, much legislation has been adopted in Europe and most countries worldwide to ban or restrict the use of chemically synthesized antibiotics in animal feed (such as growth promoters), which has triggered a need to find effective, safe and natural alternatives to antibiotics [11,12,13]. 

Mushroom polysaccharides are seen as a biological regulator with various physiological activities obtained from the mycelium of ascomycetes and basidiomycetes Subphylum mushroom (*Agaricus bisporus*, agaric, *Ganoderma lucidum*, etc.) through deep liquid fermentation. They play an important role in regulating animal immune function through stimulating natural killer cells involving neutrophils and macrophage dependent immune system responses, in addition to modifying receptors such as those of dectin-1, toll-like receptor-2, scavengers and lactosylceramides [14]. Studies on poultry have shown that mushroom polysaccharides can enhance the specific immunoglobulin level of *Eimeria tenella* infected chickens [15], stimulate the growth of immune organs such as the spleen, thymus and bursa [16], and positively modify the intestinal microbiota in infected chickens [17]. 

Nanotechnology is an emerging technology with high potential and diverse applications in agricultural sectors including environmental systems and animal feeding. It also offers potential advantages in supporting research in many areas (nanoproteomics, medicine, diagnostics, etc.) of life sciences [18,19]. Copper is one of the essential trace mineral elements in the animal body, which plays an important role in growth and development [20]. New organic copper sources have become the focus of current industry; they are better utilized than the inorganic ones, which solve problems related to using high doses of inorganic copper [21,22]. Nano-copper offers a promising solution to mediate these issues due to its different physical and chemical properties than other forms. It has been shown to have positive effects, in terms of reducing copper excretion level, improving digestible energy and absorption of fat, and enhancing antioxidant and immune function [23].

Chitosan is a poly-glucosamine made from alkaline N-deacetylation of chitin found in shells of shrimps, crabs and insects [24]. It has been known as a nuisanceless feed additive, owing to such special biological activities as lowering blood lipids, reducing body fat deposition, and improving nutritional efficacy and immune function [25,26,27]. Chitosan is soluble in acids and produces protonation of the −NH2 on the C-2 position of the d-Glucosamine repeat unit and easily forms a copper complex [28,29]. It has been demonstrated that copper loaded chitosan, as a substitute for chlortetracycline, can improve growth performance and increase immune organ indices of chickens [30]. Lysozyme is commonly found in animals, plants and microorganisms. It has been seen as a new green-healthy feed additive, nontoxic to organisms, leaving no drug residues in meat and eggs, and showing diverse biological activities [31]. Studies have indicated that lysozyme can improve the antimicrobial activity of ovo-transferrin against *Escherichia coli*, change intestinal microbiota, and improve the growth performance, gut antioxidant status, and nonspecific immunity of chickens [32,33,34]. 

Intestinal inflammatory response has been shown to cause intestinal epithelium dysfunction, and reduce the absorption of nutrients in animals, through altering the permeability of the natural barrier [35]. The reduction of intestinal inflammatory response may contribute to improved growth performance [36]. Jejunum, in particular, is the main site for digestion and absorption of nutrients in the small intestine [37]; therefore, this study investigated the intestinal inflammatory response in the jejunum segment. *Cyclooxygenase 2 (COX-2)* is one of the pro-inflammatory mediators involved in the induction of gut inflammation; a certain level of *COX-2* is critical for the maintenance of epithelial integrity, proliferation and homeostasis [38]. *Diamine oxidase (DAO)* is secreted by intestinal epithelial cells, and the increase of its activity in blood indicates the destruction of the intestinal barrier and intestinal permeability [39]. *Inducible nitric oxide synthase (iNOS)* plays an important role in the intestinal inflammatory and autoimmune response; an increased concentration of *iNOS* expresses the amount of damage in the intestinal mucosa [40]. The damaged intestinal barrier usually occurs due to changes in the expression of tight junction proteins. *Zonula occludens protein 1 (ZO-1)* is the first characterized tight junction protein; the increased expression of *ZO-1* is required for normal intestinal development [41,42]. *Mucin 2 (MUC-2)* is the first described intestinal mucin gene; abnormalities in its expression indicate the occurrence of some gastrointestinal diseases, and the decreased expression of *MUC-2* indicates higher tissue damage [43,44]. Intestinal inflammation can also increase the body temperature of the animal, making it drowsy, which reduces feed intake and ultimately leads to decreased growth performance [45,46]. The objective of this study, therefore, was to evaluate the ability of mushroom polysaccharide, nano-copper, copper loaded chitosan, and lysozyme to protect the intestinal barrier immunity of chickens under LPS stimulation.

## 2. Materials and Methods

### 2.1. Birds, Diet and Management

This study was carried out in accordance with the Animal Care Committee of the Institute of Animal Science, Guangdong Academy of Agricultural Sciences, with the approval number GAASISA-2015-036. A total of 960 healthy 4-day-old Wenshi female chicks obtained from a local hatchery (Guangdong Wiz Agricultural Science and Technology Co. Ltd, Guangzhou, China), with a similar average initial body weight (BW) of 48.73 g were randomly assigned to eight groups, each with six replicates of 20 birds (n = 120/treatment). Each replicate was housed in a floor pen (1.3 × 3.5 m) filled with wood shavings to a depth of 5 cm. Feed and water were provided *ad libitum* and natural ambient lighting was used throughout the experiment period (21 days). The eight groups were fed the basal diet supplemented with 0 (control), 0 (LPS; the birds were injected with LPS), 200 mg/kg Aureomycin (antibiotic), 50 mg/kg mushroom polysaccharide (polysaccharide 1), 100 mg/kg mushroom polysaccharide (polysaccharide 2), 500 mg/kg nano-copper, 300 mg/kg copper loaded chitosan, and 500 mg/kg lysozyme. The basal diet (Table 1) was formulated to meet the standard nutritional requirements of yellow-feathered chickens, as described in the Chinese Feeding Standard of Chicken [47]. On the 18th and the 20th day of age, birds of the control group were injected with 0.5 mL of 0.9% NaCl solution/kg BW in the breast muscle at 8:00 a.m., while the LPS and other groups were injected with an equivalent volume (0.5 mL) of saline containing 500 µg LPS/kg BW at the corresponding time. LPS was *E. coli* serotype O55: B5 (Sigma-Aldrich trading Co., Ltd, Shanghai, China). The Aureomycin antibiotic was purchased from Guangdong Newland Feed Science and Technology Co., Ltd, Guangzhou, China. Mushroom polysaccharide was purchased from Taiwan Jia Shi Kai Biotechnology Co., Ltd, Taibei, China. Nano-copper and copper loaded chitosan both were purchased from SCAU (South China Agricultural University, Guangzhou, China). Lysozyme is a microbial fermentation product (Zhejiang Aegis Biotechnology Co., Ltd, Jinhua, China). 

### 2.2. Growth Performance

The initial and final BW of the individual birds were recorded, and the average daily gain (ADG), average daily feed intake (ADFI), and feed conversion ratio (FCR) were determined on a per replicate basis between day 4 and day 24 of age. Mortality was recorded daily and was used to adjust the total number of birds per replicate to exclude them from calculations of ADFI and FCR.

### 2.3. Sampling

A total of 96 birds, two birds from each replicate (12/treatment), at day 25 were euthanized by approved methods for subsequent analyses, and exsanguinated. Blood samples were collected from the wing vein in heparinized-evacuated tubes, which were then centrifuged at 4000× *g* for 10 min at 4 °C, and plasma was kept at −80 °C until analysis. A 6-cm segment of the mid-jejunum was rinsed with phosphate-buffered saline (pH = 7.4), then mucosa was scraped with a glass slide, placed into sterile tubes, plunged into liquid nitrogen, and then stored at −80 °C until analysis. Spleen and thymus were dissected, transferred into sterile tubes after removing the connective tissue and fat, plunged into liquid nitrogen, and then stored at −80 °C for mRNA expression analysis.

### 2.4. Biochemical Indices in Plasma and Jejunal Mucosa

The plasma activities of DAO and iNOS, as well as the contents of immunoglobulin G (IgG), interferon γ (IFN-γ), and tumor necrosis factor α (TNF-α) were measured by a spectrophotometer (Biomate 5, Thermo Electron Corporation, Rochester, NY, USA). DAO and iNOS in plasma were determined using colorimetric kits (Nanjing Jiancheng Bioengineering Institute, Nanjing, China), and the IgG, IFN-γ and TNF-α were determined using chicken Elisa kits (Beijing Equation Biotechnology co., Ltd, Beijing, China). Mucosal homogenates were centrifuged at 900× *g* for 10 min at 4 °C, and the supernatants were used for biochemical assays. The contents of secretory immunoglobulin A (SIgA), IgG, immunoglobulin M (IgM), interleukin 1β (*IL-1β*), and TNF-α in jejunal mucosa were assayed using chicken Elisa kits (Beijing Equation Biotechnology co., Ltd, Beijing, China) with a spectrophotometer (Biomate 5, Thermo Electron Corporation, Rochester, NY, USA).

### 2.5. Quantitative RT-PCR (qPCR)

Total RNA was isolated from the frozen jejunal mucosa, spleen, and thymus samples by Trizol (Invitrogen, Carlsbad, CA, USA), which was then adjusted to 1000 ng/μL to synthesize a first-strand cDNA (Promega, Beijing, China). Messenger RNA was then quantified by qPCR with an ABI 7500 Real-time detection system (Applied Biosystems, Foster, CA, USA) using a SYBR^®^ Premix Ex Taq™ II kit (Takara, Dalian, China). The commercial gene primers were used based on chicken sequences (Sangon Biological Engineering Co., Ltd, Shanghai, China). In this study, we selected β-actin as the housekeeping gene for normalization purposes. The primers based on chicken sequences are listed in (Table 2). Amplification was performed in a total volume of 20 μL containing 10 μL of 2× SYBR^®^ (Bio-rad, Shanghai, China) Premix, 2 μL of 10× diluted cDNA (5 ng/μL), 1 μL of each primer (10 mmol/L), and 6 μL double distilled H_2_O. The real-time PCR program started with denaturation at 95 °C for 30 s, followed by 40 cycles at 95 °C for 10 s and 60 °C for 30 s. Dissociation analysis of the amplification products was performed after each PCR run to confirm that a single PCR product was amplified and detected. Data were analyzed with ABI 7500 SDS software (Applied Biosystems) where the baseline was set automatically by the software and average dCt values (normalized using *β-actin*) were used to calculate the relative expression levels based on the comparative Ct method calculated as 2^−ΔΔCt^. Results were expressed as relative abundances, i.e., log (2^−ΔΔCt^).

### 2.6. Statistical Analysis

A power test (Gpower Windows 3.1) was performed, based on the BW data, in order to identify the adequate number of replicates for this study. The replicate (pen) was considered as the experimental unit. Variables with non-normal distribution (as determined by the Shapiro–Wilk test; *p* < 0.05) were arcsine-transformed before analysis. Data were subjected to one-way analysis of variance using SPSS statistical software (version 19.0 for Windows; SPSS Inc.). Differences among treatment means were examined using Dunnett multiple range tests and considered significant when *p* < 0.05. Data were expressed as means and their pooled standard errors. 

## 3. Results

### 3.1. Growth Performance

The effects of treatments on growth performance traits, including final BW, ADG, ADFI, FCR, and mortality are shown in Table 3. The ADFI of all treatments was significantly lower (*p <* 0.05) than the controls, and the FCR and mortality were not affected (*p* > 0.05). 

### 3.2. Plasma and Jejunal Mucosa Biochemical Variables

The results of biochemical variables in plasma and jejunal mucosa are shown in Table 4. Compared with the results of the control group, the LPS and other treatments showed higher activities of DAO, the LPS treatment showed higher activities of *iNOS*, higher contents of *IFN-γ* and *TNF-α* in plasma, and *IL-1β* and *TNF-α* in jejunal mucosa (*p* < 0.05), but had a lower content of IgG in plasma (*p* < 0.05). Polysaccharide 2 treatment showed higher contents of SIgA and IgM, polysaccharide 2 and lysozyme treatments showed higher contents of IgG in jejunal mucosa (*p* < 0.05). Compared with LPS treatment, polysaccharide 2 treatment had lower DAO activity in plasma (*P* < 0.05), while the antibiotic, polysaccharide 2, copper loaded chitosan, and lysozyme treatments had higher IgG content in plasma (*p* < 0.05). *IFN-γ* content in plasma in all treatments were lower (*p* < 0.05), *TNF-α* content in plasma of all treatments was lower (*p* < 0.05) except for that of the nano-copper group (*p* > 0.05). The antibiotic, polysaccharide 2, and lysozyme treatments had higher SIgA content in jejunal mucosa (*p* < 0.05), IgG and IgM contents in jejunal mucosa in polysaccharide 2, and lysozyme treatments were higher (*p* < 0.05). The *IL-1β* content in jejunal mucosa in polysaccharide, and lysozyme treatments was lower (*p* < 0.05), and the content of *TNF-α* in jejunal mucosa of polysaccharide 2 and lysozyme treatments was lower (*p* < 0.05). 

### 3.3. Gene Expression in Jejunal Mucosa

The results of gene expression in jejunal mucosa are shown in Table 5. Compared with the control results, expression of *MUC-2* gene in all treatments was lower (*p* < 0.05) except for the lysozyme treatment (*p* > 0.05), and the two levels of polysaccharides showed lower expression of *iNOS* and the LPS treatment showed higher expression of *COX-2* genes (*p* < 0.05). Compared with the gene expression results obtained in the LPS treatment, dietary supplementation with antibiotic, mushroom polysaccharides 1 and 2, nano-copper, copper loaded chitosan, and lysozyme did not affect (*p* > 0.05) the expression of *ZO-1* and *MUC-2* genes, whereas the gene expression was lower with respect to that of *iNOS* in polysaccharide 2 and lysozyme treatments (*p* < 0.05), as well as that of *COX-2* in lysozyme treatment (*p* < 0.05).

### 3.4. Gene Expression in Spleen and Thymus

The results of gene expression in spleen and thymus are shown in Table 6. Compared to the results of the control, the gene expression of *TNF-α* in spleen of LPS treatment was significantly higher (*p* < 0.05), *IL-1β* in the spleen of antibiotic treatment was lower (*p* < 0.05), and *IL-1β* in the thymus of lysozyme treatment was lower (*p* < 0.05). Compared with the results of LPS treatment, a reduced gene expression was obtained in spleen *TNF-α* in polysaccharide and copper loaded chitosan treatments (*p* < 0.05), *IL-1β* in the spleen of all treatments (*p* < 0.05) except those of polysaccharide 1 and nano-copper groups (*p* > 0.05), and *IL-1β* in thymus of all treatments (*p* < 0.05) except for that of the nano-copper group (*p* > 0.05).

## 4. Discussion

The growth performance traits BW, ADG, ADFI and FCR are the primary indices reflecting the economic value of livestock and poultry. In this study (Table 3), the final BW and ADG of LPS and copper loaded chitosan treatments were significantly lower than the controls, when they were compared under Dunnett multiple tests and ensured homogenous variances, although the whole *p* value was higher than 0.05. According to the present BW data, the power test of the study (Gpower windows 3.1) showed that the power (1-β err prob) = 0.8033, and depending on the coefficient of variation, the experiment needed 48 replicates in total as a minimum number of replicates. Therefore, the number of replicates used in this study was adequate and consistent with the calculated replicate number using the Gpower test. Overall, LPS challenge here decreased the BW, ADG, and ADFI of chicks. These observations were consistent with the findings of previous studies [48,49]. Additionally, our results here revealed that the dietary supplementation with either mushroom polysaccharides, nano-copper, copper loaded chitosan, or lysozyme could not alleviate the reduction in BW, ADG, and ADFI caused by LPS. The antibiotic treatment showed the same results as those of the tested antibiotic alternatives which could not eliminate the negative effects of LPS on growth performance; the results were consistent with those of a previous study [46], and inconsistent with another [50]. The reasons for these differences might be due to the different application dose of LPS, degree of recovery from intestinal barrier damage, physical characteristics of the feed additive, or the broiler strain used in the different studies.

*DAO*, *iNOS*, *ZO-1*, *MUC-2* and *COX-2* levels were determined to evaluate the extent of damage in the intestinal tract. In this study (Table 4 and Table 5), both *DAO* and *iNOS* levels in plasma, *iNOS* and *COX-2* gene expression in jejunal mucosa were significantly increased by LPS challenge, *MUC-2* gene expression in jejunal mucosa were significantly decreased by LPS challenge; these results were consistent with findings of previous studies [46,51,52], which indicate that LPS have a significant role in damaging the function of the intestinal mucosal barrier. The present data also showed that the tested antibiotic alternatives have different beneficial effects on the intestinal barrier. The dietary addition of 100 mg/kg mushroom polysaccharide reduced the level of DAO in plasma (Table 4); this result shows consistency with the application of modified clinoptilolite in broilers [46]. Dietary addition of mushroom polysaccharide (100 mg/kg) and lysozyme decreased the gene expression of *iNOS* in jejunal mucosa (Table 5); the result was comparable to those obtained by the application of methanol extract of *Zanthoxylum rhetsa* in RAW 264.7 macrophages [53]. The tested lysozyme decreased the gene expression of *COX-2* in jejunal mucosa (Table 5), and this result was comparable to the application of forsythiaside in chickens [54]. The results here, therefore, proved that dietary mushroom polysaccharide (100 mg/kg) and lysozyme reduced the jejunum barrier damage caused by LPS. These positive effects may be related to the fact that mushroom polysaccharide (100 mg/kg) decreased the content of *DAO* in plasma and the expression of *iNOS* in jejunal mucosa; and lysozyme decreased the gene expression of *COX-2* in jejunal mucosa.

Specific immunity is the third defense line of animal immunity, and it is mainly composed of immune organs and immune cells; lymphocyte B cells are responsible for humeral immunity and T cells are responsible for cellular immunity. In general, IgG and IgM levels are the indices used to examine immune function; the reduced levels indicate immune deficiency [55,56]. In this study (Table 4), LPS challenge significantly decreased IgG content in plasma, and IgG and IgM in jejunal mucosa compared to the controls, which implies negative effects of LPS on the immunity. The results here showed that the dietary addition of aureomycin, mushroom polysaccharide (100 mg/kg), copper loaded chitosan, and lysozyme increased IgG content in plasma, and dietary addition of mushroom polysaccharide (100 mg/kg) and lysozyme increased contents of IgG and IgM in jejunal mucosa; these observations were consistent with previous findings [57]. SIgA as main immunoglobulin is the first line of defense in the intestinal mucosa, expressed constitutively in the intestine and usually associated with increased innate immune defense. It can resist various endogenous symbiotic bacteria and exogenous pathogens, its level reflects the body’s immunity [58]. In this study, LPS challenge had no significant effect on SIgA content in jejunal mucosa. The dietary addition of Aureomycin, mushroom polysaccharide (100 mg/kg), and lysozyme significantly increased SIgA content jejunal mucosa; similar results were obtained with the application of L-theanine in yellow-feathered broilers [50]. The most important finding in the present study, is that substituting dietary antibiotic with mushroom polysaccharide (100 mg/kg), copper loaded chitosan and lysozyme alleviated the reduction in immunity caused by LPS through increasing the level of immunoglobulins.

The secretion of cytokines is an important host defense mechanism following bacterial infection, and their intensity is one of the major evaluation factors of bacterial pathogenicity [59]. IFN-γ, *TNF-α* and *IL-1β* have long been recognized as signature pro-inflammatory cytokines that play a central role in inflammation and autoimmune diseases, high levels are positively correlated with disease outbreak [60,61]. In this study (Table 4 and Table 6), the LPS challenge significantly increased the content of *IFN-γ* and *TNF-α* in plasma and *IL-1β* and *TNF-α* in jejunal mucosa (Table 4). Similar changes in gene expression of *TNF-α* in the spleen were observed in LPS treatment (Table 5). These results are consistent with previous reports, which indicated that LPS induce the production and expression of proinflammatory cytokines [62,63,64]. Except for antibiotic, nano-copper, and copper loaded chitosan treatments, the tested dietary additives here decreased the content of *IFN-γ* in plasma, and *IL-1β* content in jejunal mucosa. The tested feed additives, except for nano-copper, significantly decreased the contents of *TNF-α* in plasma and gene expression of *IL-1β* in thymus. The dietary addition of Aureomycin, mushroom polysaccharide (100 mg/kg), copper loaded chitosan, and lysozyme significantly decreased gene expression of *IL-1β* in the spleen, and mushroom polysaccharide (100 mg/kg) and lysozyme had similar effects on *TNF-α* content in jejunal mucosa. The dietary addition of mushroom polysaccharide and lysozyme significantly decreased gene expression of spleen *TNF-α* than that which occurred in LPS treatment; these observations were consistent with previous findings [46,54], and suggest that the dietary antibiotic alternatives of mushroom polysaccharide (100 mg/kg), copper loaded chitosan and lysozyme improved the immunity by reducing the concentrations and gene expression of proinflammatory cytokines induced by LPS.

## 5. Conclusions

In conclusion, the tested antibiotic alternatives here have proven beneficial effects on alleviating several negative effects of LPS on jejunal barrier and immunity. The dietary addition of mushroom polysaccharides (50 mg/kg) reduced the contents of *IFN-γ* and *TNF-α* in plasma, *IL-1β* in jejunal mucosa, gene expression of *TNF-α* in spleen and *IL-1β* in thymus. Dietary mushroom polysaccharides (100 mg/kg), as well as lysozyme (500 mg/kg) reduced activities of *DAO*, contents of *IFN-γ* and *TNF-α* in plasma, contents of *IL-1β* and *TNF-α* in jejunal mucosa, increased contents of SIgA, IgG and IgM in plasma and jejunal mucosa, reduced gene expression of *iNOS* in jejunal mucosa, reduced *TNF-α* and *IL-1β* in spleen and *IL-1β* in thymus. The dietary addition of nano-copper (500 mg/kg) reduced content of IFN-γ in plasma, and the copper loaded chitosan (300 mg/kg) reduced the contents of IFN-γ and *TNF-α* in plasma, and reduced gene expression of *TNF-α* and *IL-1β* in the spleen and *IL-1β* in the thymus. The tested antibiotic alternatives in this study did not alleviate the negative effects of LPS on BW, ADG and ADFI of yellow-feathered female chickens aged 4 to 24 days; they showed similar results to those obtained by the antibiotic treatment. 

## Figures and Tables

**Table 1:** Composition and nutrient levels of the experimental diets (air-dry basis, %) of yellow-feathered chickens aged 4 to 24 days.

Item	Basal Diet
Ingredients (%)	
Corn	57.60
Soybean meal	34.20
Soybean oil	2.10
DL-Methionine	0.15
Limestone	1.20
Monocalcium phosphate	1.90
NaCl	0.30
Corn cob meal	1.55
Premix ^1^	1.00
Total kg	100.00
Nutrient contents ^2^	
AME (MJ/kg) ^3^	12.14
Crude Protein %	21.00
Lysine%	1.16
Methionine %	0.46
Calcium %	1.00
Available Phosphorus %	0.44

^1^ The vitamins and minerals in the diet were supplied exactly as stated by the Chinese Feeding Standard of Chicken (2004). To provide the following per kilogram of diet: vitamin A, 15 000 IU; vitamin D_3_, 3 300 IU; vitamin E, 10 IU; vitamin K_3_, 5 mg; thiamin, 1.8 mg; riboflavin, 3.6 mg; pyridoxine, 3.5 mg; cyanocobalamin, 0.01 mg; calcium pantothenic, 10 mg; niacin, 35 mg; folic acid, 0.55 mg; biotin, 0.15 mg; choline chloride, 1000 mg; Fe, 80 mg; Cu, 8 mg; Mn, 80 mg; Zn, 60 mg; I, 0.35 mg; Se, 0.15 mg. The carrier was corn cob meal. ^2^ Values were calculated from data provided by Feed Database in China (2016). ^3^ Apparent metabolic energy.

**Table 2:** Sequences of real-time PCR primers.

Gene Name	Sequence	GenBank No.
*β-actin*	F-5′-GAGAAATTGTGCGTGACATCA-3′	NM_205518
	R-5′-CCTGAACCTCTCATTGCCA-3′	
*ZO-1* ^1^	F-5′-CCAAAGACAGCAGGAGGAGA-3′	XM_015278981.1
	R-5′-TGGCTAGTTTCTCTCGTGCA-3′	
*MUC-2* ^2^	F-5′-CATTCAACGAGGAGAGCTGC-3′	NM_001318434.1
	R-5′-TTCCTTGCAGCAGGAACAAC-3′	
*iNOS* ^3^	F-5′-AGCATAACTCCCGTGTTCCA-3′	NM_204961.1
	R-5′-GATTTCCCAGTCTCGGTTGC-3′	
*COX-2* ^4^	F-5′-TGCAACGATATGGCTGAG-3′	YP_009558655.1
	R-5′-CTGCGGATTCGGTTCTGGTAT-3′	
*TNF-α* ^5^	F-5′-GAAGCAGCGTTTGGGAGTG-3′	NM_204267.1
	R-5′-GTTGTGGGACAGGGTAGGG-3′	
	R-5′-CAGGTCGCTGTAGGAATTGC-3′	
*IL-1β* ^6^	F-5′-GAAGTGCTTCGTGCTGGAGT-3′	NM_204524.1
	R-5′-ACTGGCATCTGCCCAGTTC-3′	
*IFN*-*γ*^7^	F-5′-GCCGCACATCAAACACATATCT-3′	NM_205427.1
	R-5′-TGAGACTGGCTCCTTTTCCTT-3′	

^1^ Zonula occludens protein 1; ^2^ Mucin 2; ^3^ Inducible nitric oxide synthase; ^4^ Cyclooxygenase 2; ^5^ Tumor necrosis factor α; ^6^ Interleukin 1β; ^7^ Interferon γ.

**Table 3:** Effects of mushroom polysaccharide, nano-copper, copper loaded chitosan, and lysozyme on growth performance of yellow-feathered chickens challenged with lipopolysaccharides (LPS).

Variables	Treatments								SEM ^2^	*p*-Value
	Control	LPS ^1^	Antibiotic	Polysaccharide 1	Polysaccharide 2	Nano-copper	Copper Loaded Chitosan	Lysozyme		
IBW ^3^ (g)	48.7	48.7	48.7	48.7	48.7	48.7	48.7	48.7	<0.01	0.990
FBW ^4^ (g)	405.2^A^	375.7^B^	384.2	381.0	388.8	385.1	377.6^B^	389.0	2.45	0.074
ADG ^5^ (g)	17.0^A^	15.6^B^	16.0	15.8	16.2	16.0	15.7^B^	16.2	0.12	0.074
ADFI ^6^ (g)	31.4^A^	28.8^B^	28.8^B^	29.0^B^	29.7^B^	29.8^B^	29.2^B^	29.8^B^	0.17	<0.001
FCR ^7^	1.85	1.85	1.80	1.84	1.84	1.86	1.87	1.84	0.01	0.21
Mortality (%)	0.00	1.67	0.00	0.83	0.00	0.83	0.00	0.00	0.22	0.47

Capital letters indicate statistically significant (*p* < 0.05) differences between control and other treatments by Dunnett tests; small letters indicate statistically significant (*p* < 0.05) differences between LPS group and the other treatments. ^1^ birds were fed the control diet and injected with LPS; ^2^ Standard error of the mean (n = 6 pens); ^3^ Initial body weight; ^4^ Final body weight; ^5^ Average daily gain; ^6^ average daily feed intake; ^7^ Feed conversion ratio.

**Table 4:** Effects of mushroom polysaccharide, nano-copper, copper loaded chitosan, and lysozyme on plasma and jejunal mucosa biochemical variables of yellow-feathered chickens challenged with lipopolysaccharides (LPS).

Variables	Treatments								SEM ^2^	*p*-Value
	Control	LPS ^1^	Antibiotic	Polysaccharide 1	Polysaccharide 2	Nano-Copper	Copper Loaded Chitosan	Lysozyme		
Plasma										
*DAO*^3^ (U/L)	0.59 ^B^	1.72 ^Aa^	1.72 ^A^	1.54 ^A^	1.33 ^Ab^	1.54 ^A^	1.60 ^A^	1.57 ^A^	0.05	<0.001
*iNOS*^4^ (U/mL)	9.97 ^B^	13.25 ^A^	11.03	11.51	11.36	11.97	11.55	9.43	0.28	0.022
*IgG*^5^ (µg/mL)	745.99 ^A^	560.11 ^Bb^	709.71 ^a^	643.24	797.36 ^a^	644.22	762.82 ^a^	712.52 ^a^	15.28	0.001
*IFN-γ*^6^ (ng/L)	54.30 ^B^	64.19 ^Aa^	52.66 ^b^	54.67 ^b^	48.99 ^b^	49.20 ^b^	49.55 ^b^	47.76 ^b^	0.79	<0.001
*TNF-α*^7^ (ng/L)	63.92 ^B^	78.24 ^Aa^	64.54 ^b^	63.49 ^b^	60.41 ^b^	72.20	59.80 ^b^	50.00 ^Bb^	1.29	<0.001
Jejunal mucosa										
*SIgA*^8^ (µg/mg)	31.80 ^B^	28.63 ^b^	40.88 ^a^	31.91	46.59 ^Aa^	35.61	31.04	37.77 ^a^	1.10	<0.001
*IgG* (µg/mg)	237.61 ^B^	222.88 ^b^	274.87	232.08	368.97 ^Aa^	279.90	295.07	336.15 ^Aa^	9.60	<0.001
*IgM*^9^ (µg/mg)	31.61 ^B^	25.33 ^b^	29.96	31.08	38.04 ^Aa^	28.29	35.78	37.53 ^a^	1.03	0.014
*IL-1β*^10^ (ng/g)	5.77 ^B^	8.60 ^Aa^	7.00	5.94 ^b^	5.87 ^b^	6.62	6.90	6.48 ^b^	0.21	0.013
*TNF-α* (ng/g)	14.63 ^B^	23.65 ^Aa^	18.77	20.36	15.56 ^b^	18.37	17.72	14.29 ^b^	0.71	0.009

Capital letters indicate statistically significant (*p* < 0.05) differences between control and other treatments by Dunnett test; small letters indicate statistically significant (*p* < 0.05) differences between LPS group and the other treatments. ^1^ birds were fed the control diet and injected with LPS; ^2^ Standard error of the mean (n = 6 pens); ^3^ Diamine oxidase; ^4^ Inducible nitric oxide synthase; ^5^ Immunoglobulin G; ^6^ Interferon γ; ^7^ Tumor necrosis factor α; ^8^ Secretory immunoglobulin A; ^9^ Immunoglobulin M; ^10^ Interleukin 1β.

**Table 5:** Effects of mushroom polysaccharide, nano-copper, copper loaded chitosan, and lysozyme on gene expression in jejunal mucosa of yellow-feathered chickens challenged with lipopolysaccharides (LPS).

Variables	Treatments								SEM ^2^	*p*-Value
	Control	LPS ^1^	Antibiotic	Polysaccharide 1	Polysaccharide 2	Nano-Copper	Copper Loaded Chitosan	Lysozyme		
*ZO-1* ^3^	1.01	0.69	0.94	0.66	0.87	0.98	1.04	1.09	0.04	0.053
*MUC-2* ^4^	1.08 ^A^	0.26 ^B^	0.54 ^B^	0.47 ^B^	0.57 ^B^	0.36 ^B^	0.38 ^B^	0.65	0.05	0.001
*iNOS* ^5^	0.94 ^A^	1.49 ^a^	1.14	1.06	0.60 ^Bb^	1.26	1.27	0.77 ^b^	0.07	0.013
*COX-2* ^6^	1.10 ^B^	2.26 ^Aa^	1.86	1.48	1.04	1.03	1.18	0.63 ^b^	0.13	0.026

Capital letters indicate statistically significant (*p* < 0.05) differences between the control and other treatments by Dunnett test; small letters indicate statistically significant (*p* < 0.05) differences between LPS group and the other treatments. ^1^ birds were fed the control diet and injected with LPS; ^2^ Standard error of the mean (n = 6 pens); ^3^ Zonula occludens protein 1; ^4^ Mucin 2; ^5^ Inducible nitric oxide synthase; ^6^ Cyclooxygenase 2.

**Table 6:** Effects of mushroom polysaccharide, nano-copper, copper loaded chitosan, and lysozyme on gene expression in spleen and thymus of yellow-feathered chickens challenged with lipopolysaccharides (LPS).

Variables	Treatments								SEM ^2^	*p*-Value
	Control	LPS ^1^	Antibiotic	Polysaccharide 1	Polysaccharide 2	Nano-Copper	Copper Loaded Chitosan	Lysozyme		
Spleen										
*IFN-γ* ^3^	0.88	1.43	1.16	0.79	0.93	0.95	0.75	1.69	0.10	0.119
*TNF-α* ^4^	1.05 ^B^	1.87 ^Aa^	1.50	1.04 ^b^	0.97 ^b^	1.37	0.86 ^b^	1.32	0.07	0.001
*IL-1β* ^5^	1.27 ^A^	1.84 ^a^	0.55 ^Bb^	1.23	0.78 ^b^	1.16	0.77 ^b^	1.02 ^b^	0.10	0.030
Thymus										
*IFN-γ*	1.04	1.18	1.41	1.40	0.98	1.47	1.36	1.55	0.09	0.682
*TNF-α*	1.01	1.38	0.94	1.30	0.98	1.55	1.18	1.11	0.06	0.119
*IL-1β*	1.13 ^A^	1.68 ^a^	1.04 ^b^	1.10 ^b^	0.96 ^b^	1.32	1.06 ^b^	0.90 ^Bb^	0.06	0.042

Capital letters indicate statistically significant (*p* < 0.05) differences between the control and other treatments by Dunnett test; small letters indicate statistically significant (*p* < 0.05) differences between LPS group and the treatment groups. ^1^ birds were fed the control diet and injected with LPS; ^2^ Standard error of the mean (n = 6 pens); ^3^ Interferon γ; ^4^ Tumor necrosis factor α; ^5^ Interleukin 1β.
